# Tattoo discrimination in Mexico motivates interest in tattoo removal among structurally vulnerable adults

**DOI:** 10.3389/fpubh.2022.894486

**Published:** 2022-08-18

**Authors:** Victoria D. Ojeda, Christopher Magana, Omar Shalakhti, Adriana Carolina Vargas-Ojeda, Jose Luis Burgos

**Affiliations:** ^1^Herbert Wertheim School of Public Health, University of California, San Diego, San Diego, CA, United States; ^2^Department of Medicine, School of Medicine, University of California, San Diego, San Diego, CA, United States; ^3^Facultad de Medicina y Psicología, Universidad Autónoma de Baja California, Tijuana, Baja California, Mexico

**Keywords:** Mexico, tattoo removal, tattoo stigma, migration, deportation

## Abstract

Tattoos are less prevalent in Mexico and tattooed persons are frequently stigmatized. We examine the prevalence and correlates of interest in receiving tattoo removal services among 278 tattooed Mexican adults living in Tijuana, Mexico who responded to interviewer-administered surveys, including open-ended questions. Overall, 69% of participants were interested in receiving free tattoo removal services, 31% reported facing employment barriers due to their tattoos, and 43% of respondents regretted or disliked some of their tattoos. Having a voter identification card, reporting moderate/severe depression symptoms and believing that tattoo removal would remove employment barriers were independently associated with interest in tattoo removal. Our findings suggest that there is substantial interest in tattoo removal services. Publicly financed tattoo removal services may help disadvantaged persons gain access to Mexico's labor market and it may positively impact other life domains such as mental well-being and interactions with law enforcement.

## Introduction

Tattooing is practiced around the world and is considered a form of art and body modification (1). Tattoos have been used as a form of self-expression, during rites of passage, to convey information about relational ties among community subgroups and about the tattooed individual; tattoos have also been used as a form of punishment (1). Over the past several decades, tattoos have gained in popularity in the United States (U.S.) and elsewhere (2). In Mexico, an estimated 12 million individuals are tattooed (3). However, tattoos may be associated with anti-social behaviors and tattooed individuals may experience negative reactions from the community (4–10). Tattoo-related stigma may create additional barriers for resettlement, among the thousands of migrants deported from the U.S. (i.e., deportees), many of whom settle in the Mexican border city of Tijuana which lies adjacent to California, U.S. (11, 12). For example, tattooed adults living in Tijuana have reported discrimination in employment, housing, as well as negative interactions with local law enforcement due to their tattoos (13–16). This study examines interest in receiving tattoo removal among structurally vulnerable adults in Tijuana, as this service may help reduce stigma experienced by tattooed community members.

Deportees residing in Tijuana face a unique risk environment that can challenge their emotional, physical and social well-being (13, 15–19). In addition, politicians and law-enforcement agencies in the U.S. have portrayed undocumented immigrants as a threat to public safety (20–23) influencing Mexicans' perceptions of deportees (9, 15, 24, 25). In Mexico, deportees often face discrimination from community members and the police, who may view them as criminals (13–16, 19). Stigma associated with tattoos may exacerbate the social precarity and vulnerability experienced by deportees in Mexican communities (4, 7–9, 15, 16).

Stigma is recognized to be a socially constructed concept that is characterized by multiple dimensions. Goffman's pioneering work initially documented the ways in which community members' treatment and perception of individuals may vary when individuals' characteristics deviate from what is deemed to be expected and the norm (26). Stigma is thus conceptualized as being created when an individual has a visible or non-visible undesirable trait that modifies an individual's relationship with other community members. Stereotyping may occur because of perceived or actual differences and the affected person's status in society may be adversely impacted (26). Additional work by Link and Phelan advance our understanding of stigma by highlighting the influence of institutions and other power structures, such as policies, in supporting the stigmatization and exclusion of individuals or groups of individuals who are deemed to not conform to the broader society's norms (26, 27). Stigma has been found to extend to the affected individual resulting in self-stigma and to that person's close contacts through stigma by association (28, 29). A growing body of work has recognized that interpersonal and structural discrimination can adversely influence health outcomes and well-being including self-esteem and self-efficacy (27, 30).

Tattoos may be stigmatized when they are visible or contain markers of stigmatized affiliations or images that are viewed as being anti-social (e.g., gang symbols) (1, 31–33). Individuals with tattoos placed near their face or hands may be judged to be of poor character (6, 34, 35), discriminated against by employers (6, 36), or harassed by police (16). Tattoo-related stigma may create feelings of regret, lead some to hide their tattoos in order to avoid discrimination or generate an interest in tattoo removal (15, 16, 37–41). While laser tattoo removal is effective (42), it is also a financially burdensome and time consuming procedure (43). The prohibitive costs of professional tattoo removal services may lead some individuals to resort to amateur methods to remove their tattoos (1, 44), which are often ineffective and can have harmful side effects including pain and scarring (38, 45, 46).

In the United States, a limited number of free or subsidized tattoo removal programs for structurally vulnerable populations (e.g., former gang members, probationers) are available (14, 44, 47). Laser tattoo removal may aid in reducing social stigma, improve social relationships, improve labor market participation, and improve the well-being of structurally vulnerable populations (44, 48, 49). However, less is known about the experiences or characteristics of tattooed Mexicans, including migrants and deportees, the prevalence of tattoo regret, interest in receiving tattoo removal, and reasons for seeking this service. These topics are the focus of this investigation which was conducted with a large sample of economically disadvantaged tattooed Mexican adults in Tijuana, Mexico; a large proportion of whom are migrants. We hypothesized that tattooed deported migrants and unemployed persons would be most interested in undergoing laser tattoo removal. Analyses were stratified by participants' interest in receiving tattoo removal in order to shed light on the characteristics of those who believe they may benefit from this service. Findings can inform the implementation of programs to support tattooed persons' integration into society and may have relevance for other communities where tattooed persons are stigmatized (50–52).

## Methods

### Participants and data collection

This mixed-methods cross-sectional study is based on data collected between January-May 2013. A convenience sample of 584 Mexican adults ages 18+ participated in the study; persons who were younger than age 18 based on self-report or who could not provide informed consent were excluded from joining the study. This analysis is limited to 278 tattooed Mexican adults (47% of the full sample, data not shown) attending a free healthcare clinic in Tijuana's *Zona Norte* [red light district] <1 mile of the U.S.-Mexico border.

In brief, participants responded to an interviewer-administered questionnaire (15, 16) designed to understand the health and social needs of disadvantaged persons in the region, including migrants (i.e., deported, internal, and cross-national migrants). Eligibility criteria for this analysis were: (1) Mexico-born age ≥18 years old; (2) seeking any service at the study site; (3) speaking Spanish or English, and 4) having ≥1 tattoo. All participants who met these criteria were invited to participate; those interested in joining the study provided their signed informed consent and received refreshments and $10 compensation for their time. This study was approved by the University of California, San Diego Human Research Protection Program and the Ethics Boards of the Health Frontiers in Tijuana Clinic and the Autonomous University of Baja California Medical School.

### Measures

#### Quantitative data

The survey was developed by the researchers, with the exception of the depression scale, for application to this unique setting; it has been used to support prior research (15, 16). Trained bilingual interviewers administered the survey. Data collection lasted ~45 min and interviewers entered participants' responses in tablet computers utilizing Qualtrics survey software (Provo, UT, US). *Socio-demographic* factors included age, gender, and U.S. migrant status (never migrated; deported migrant; non-deported migrant). *Risk Environment measures* consider the following conditions: recent drug use or injection drug use (i.e., past 6 months; both yes/no), recent trading sex (past 6 months), ever incarcerated in the USA or Mexico or both countries (yes/no). *Social exclusion* variables included: possession of a Mexican federal voter identification card (yes/no), covered by *Seguro Popular* (yes/no) a federal public health insurance program which covers impoverished persons (53), and depression symptoms (none to mild vs. moderate to severe) per the Patient Reported Outcomes Measurement Information System (PROMIS) depression short form (PROMIS-D-8; 8b short form) (54, 55). Recent homeless status was defined by where participants slept most frequently in the prior 6 months: those who slept in migrant shelters, churches, streets, public parks, vacant lots, or the Tijuana River canal were classified as homeless. Participants responded to diverse adverse encounters: “During the last 6 months… a) have you ever been threatened or harassed by police, federal agents or army members in Tijuana? b) denied a job in Tijuana, c) denied access to housing or a shelter or other place that you can sleep or live in Tijuana? Respondents also identified potential access to social support, responding to the question: “Do you have friends or family in Tijuana”? (yes/no). These data are shown in Table 1.

**Table 1:** Sociodemographic characteristics of Mexican tattooed adults (*n* = 278) receiving free medical care, stratified by interest in receiving free laser tattoo removal at the study site, Tijuana, Mexico, 2013.

			**Interested in free tattoo removal at clinic**				
	**Overall** **sample** **(*****n*** = **278, 100%)**		**No, not** **interested** **(*****n*** = **85, 31%)**		**Yes**, **interested** **(*****n*** = **193, 69%)**		
**Sociodemographics**	**%**	***N***	**%**	***N***	**%**	***N***	***p*-value**
Age							
18 to 36	37%	103	41%	35	35%	68	0.63
37 to 47	40%	110	36%	31	41%	79	
≥48	23%	65	22%	19	24%	46	
Gender							
Female	21%	59	16%	14	23%	44	0.20
Male	79%	219	84%	71	77%	149	
US migrant status							
Never migrated to U.S.	15%	43	8%	7	19%	36	0.09
Deported US migrant	67%	186	73%	62	64%	124	
Non-deported US migrant	18%	49	19%	16	17%	33	
Risk environment							
Recent drug use^a^	**60%**	**168**	**69%**	**59**	**56%**	**109**	**0.04**
Recent injection drug use^a^	**36%**	**99**	**45%**	**38**	**32%**	**61**	**0.04**
Recently traded sex^a^	17%	48	15%	13	18%	35	0.56
Ever incarcerated (Mexico, USA, or both)	80%	221	79%	67	80%	154	0.85
Social exclusion							
IFE Mexican voter identification card	**37%**	**104**	**25%**	**21**	**43%**	**83**	**<0.01**
Has *Seguro Popular* (Public Health Insurance)	**33%**	**93**	**25%**	**21**	**37%**	**72**	**0.04**
Has moderate to severe depression	**34%**	**94**	**24%**	**20**	**38%**	**74**	**0.02**
Recently homeless^a^	53%	146	56%	48	51%	98	0.38
Threatened by law enforcement in Tijuana^a^	49%	135	46%	39	50%	96	0.55
Denied employment in Tijuana^a^	44%	122	36%	31	47%	91	0.10
Denied housing in Tijuana^a^	17%	48	15%	13	18%	35	0.56
Lacks friends or family in Tijuana	**67%**	**185**	**64%**	**54**	**68%**	**131**	**0.48**

Percentages may not sum to 100% due to rounding; comparisons which are significant at p < 0.05 are denoted in bold type.

a Reflects a behavior or experience occurring in the 6 months prior to study participation.

We characterize participants' tattoos (Table 2), including the total number of tattoos (1; 2–3; ≥4), tattoo visibility (forearms, hands, face, neck; versus not on these locations), tattoo imagery/content (text/names; animals/nature-images; religious images; death/skulls; weapons/gang symbols). Participants reported their feelings about the tattoos (i.e., does not like some or all tattoos vs. likes all tattoos or is indifferent about them), whether they believe that they have experienced barriers to employment because of their tattoos (yes/no), and beliefs that removing tattoos would reduce barriers to employment (yes/no).

**Table 2:** Tattoo characteristics and participants' perceptions, reported by Mexican tattooed adults (*n* = 278) receiving free medical care, stratified by interest in receiving free laser tattoo removal at the study site, Tijuana, Mexico, 2013.

			**Interested in free tattoo removal at clinic**				
	**Overall** **sample** **(*****n*** = **278, 100%)**		**No, not** **interested** **(*****n*** = **85, 31%)**		**Yes**, **interested** **(*****n*** = **193, 69%)**		
**Tattoo characteristics**	**%**	***N***	**%**	***N***	**%**	***N***	***p*-value**
Number of tattoos							
1 tattoo	27%	75	33%	28	24%	46	0.29
2–3 tattoos	30%	83	27%	23	31%	60	
≥ 4 tattoos	44%	122	40%	34	46%	89	
Has visible tattoos	37%	102	34%	29	38%	73	0.56
Tattoo imagery/content^a^							
Text or names	70%	195	69%	59	70%	136	0.86
Animals or nature	32%	89	31%	26	33%	63	0.74
Religious	17%	47	19%	16	16%	31	0.57
Death or skulls	12%	32	11%	9	12%	23	0.75
Weapons or gang symbols	8%	22	7%	6	8%	16	0.73
Participant perceptions							
Participant does not like some or all tattoos	**43%**	**120**	**6%**	**5**	**60%**	**115**	**<0.01**
Has experienced barriers to employment because of tattoos	**31%**	**85**	**20%**	**17**	**35%**	**68**	**0.01**
Believes removing tattoos will aid in finding employment in Tijuana	**56%**	**151**	**41%**	**34**	**62%**	**117**	**<0.01**

Percentages may not sum to 100% due to rounding; comparisons which are significant at p < 0.05 are denoted in bold type.

a Categories are not mutually exclusive and may sum to >100%.

For the dependent variable, participants were asked: “Imagine that in the next 6 months there was a free service here [at the clinic] to remove tattoos, do you think you would be interested in using those services?” (yes/no).

#### Qualitative data derived from the survey

The questionnaire included several open-ended questions. Participants interested in removing some or all of their tattoos were asked: “*Currently, what are all the reasons for which you DO want to remove your tattoos*?” Interviewers entered participants' responses (*n* = 156) for those who responded in the affirmative into the survey software verbatim.

#### Analysis

Descriptive statistics were generated using STATA v16 to characterize participants' sociodemographic, tattoo-related, and vulnerability characteristics; analyses were stratified by interest in receiving free tattoo removal at the clinic where the study was conducted (Tables 1, 2). For categorical variables, we employed Pearson chi-square tests to assess statistical significance between groups. Variables attaining significance levels of *p* < 0.10 in binary analyses were considered for inclusion in multivariable logistic regression models that assessed the relationship between each independent variable and interest in receiving free tattoo removal services at the clinic. We controlled for migrant status given the pervasiveness of tattoos among U.S. migrants (**Table 4**).

Qualitative text data were entered into a spreadsheet and two authors utilized the methodology of “Coding Consensus, Co-occurrence, and Comparison,” based on grounded theory techniques (56, 57) to code responses and identify emergent themes; conflicts in coding were discussed and resolved (56). Some responses were assigned multiple codes. The main themes are described and illustrative quotes are provided in English and Spanish (Table 3). The authors translated all quotes into English. We provide percentages for each theme to illustrate its significance within the text responses (58). Participants who indicated that they did not want to remove their tattoos were asked why they did NOT want to remove their tattoos and themes emerging from participants' responses (*n* = 89) are summarized in the text (data not shown in table). The responses to both questions represent 245 responses (i.e., 88% of tattooed participants); participants were not required to respond to these questions, though most did.

**Table 3:** Reasons for interest in receiving tattoo removal expressed by Mexican tattooed adults (*n* = 156) receiving free medical care, Tijuana, Mexico, 2013.

	**Spanish Language Quote**	**English Translation of Quote**
Employment barriers (*n* = 48; 31%)	4. [Porque] no me dan trabajo por tener tatuajes	4. [Because] they don't give [me] any work because I have tattoos
	20. Por dificultad para conseguir trabajo.	20. Due to difficulties in getting a job.
	77. Porque en muchos lugares no puede trabajar con los tatuajes	77. Because in many places you can't work with tattoos
	43. Porque se me complica conseguir empleo y se miran mal	43. Because it becomes more difficult to get a job and they [tattoos] look bad
	30. Me siento orgulloso de él [tatuaje] porque es el nombre de mi hija, pero me gustaría quitármelo porque me ocasiona problemas para buscar trabajo	30. I feel proud of it [the tattoo] because it is my daughter's name, but I would like to remove it because it creates problems in finding a job
Do not like/regret their tattoos	9. Me arrepiento, eran momentos de mi juventud	9. I regret [it], they were moments of my youth
(*n* = 44; 28%)	23. Ya siento que no son apropiados, no me sirven para nada, ya no tienen un significado para mi	23. I now feel that they are not appropriate, they are useless to me, they no longer have meaning to me
	114. Ya no me gustan, estoy arrepentido de habérmelos puesto	114. I don't like them anymore, I regret having gotten them [tattoos]
Tattoos make a bad impression (*n* = 36; 23%)	15. Porque me marcaron como persona peligrosa por el tatuaje	15. Because they labeled me as a dangerous person because of the tattoo
	24. Me dan mala imagen	24. They make a bad impression
	86. Porque tengo un niño chiquito–no me gusta que me lo vea, no quisiera que él se hiciera un tatuaje, y para que me acepten en los trabajos	86. Because I have a little boy–I don't like him to see me with it [tattoo], I would not want him to get a tattoo, and for me to be accepted for jobs
	113. Para tener mejor aspecto hacia la sociedad porque a veces lo miran a uno feo por los tatuajes	113. To make a better impression toward society, because at times, they look at you badly because of the tattoos
	119. Porque no son buenos, no puede uno encontrar trabajo, no son necesarios, discriminan a las personas con tatuajes, piensan que es ratero, pandillero	119. Because they [tattoos] are not good, one cannot find work, they are not necessary, they discriminate against people with tattoos, they think he is a thief, gang member
	175. Porque ya se ven muy feos, y está uno muy grande y le da a uno vergüenza, y ninguno de mis hijos están rayados y es como una [mala] carta de recomendación	175. Because they look very ugly now, and when a person is very old they are embarrassing, and none of my children are inked and it's like a [bad] letter of recommendation
	187. La gente discrimina a los tatuajes, y se ve mal.	187. People discriminate against tattoos, and it looks bad.
Thinks tattoos are ugly (*n* = 29; 19%)	5. Porque no me gusta como se me ve-fue un tatuaje casero	5. Because I don't like how it looks on me- it was a homemade tattoo
	39. No me gustan	39. I don't like them [the tattoos]
	63. Porque no quedó bien	63. Because it didn't come out right
Think life would be better without tattoos (*n* = 25; 16%)	60. Porque me afectan en muchas maneras, no puedo encontrar trabajos, me ocasionan problemas personales y familiares	60. Because they affect me in many ways, I can't find jobs, they create personal and family problems for me
	79. Porque veo a las demás personas y se me hacen ridículos y yo no me quiero ver así	79. Because I see other [tattooed] people and I think they look ridiculous, and I don't want to look like that
	124. Para vivir mejor y encontrar trabajo	124. To live better and find a job
	141. No me gusta como me trata la gente	141. I don't like how people treat me
Mental health impact (*n* = 25; 16%)	25. No me siento a gusto con el	25. I don't feel comfortable with it [the tattoo]
	38. Por diferentes cosas, porque pienso que ya se miran mal, me agüito cuando voy a mi cantón con mi familia	38. For different things, because I think they look bad now, I get sad when I go to my home with my family
	73. Me dan vergüenza porque me los hicieron sin que me diera cuenta a los 13 años	73. I am ashamed of them because they put them [tattoos] on me without my knowledge when I was 13 years old
	103. Simplemente me gustaría estar limpio	103. I would simply like to look clean
	128. Uno se cansa de la discriminación	128. One gets tired of discrimination
	163. Pues porque me da pena saludar y se ven	163. Well, because I feel embarrassed to say hi [to people] and they [the tattoos] can be seen
Prevent police interactions (*n* = 11; 7%)	16. Porque hay mucha molestia con la policía	16. Because there is a lot of trouble with the police
	57. Llama mala atención con la ley	57. Attracts bad attention from law enforcement
	65. Me identifican muy rápido los policías	65. The police identify me very quickly [because of the tattoos]

## Results

### Participant characteristics

Table 1 presents sociodemographic characteristics and exposure to the risk environment among a sample of tattooed Mexicans (*n* = 278), stratified by interest in receiving free tattoo removal at the study site. Overall, 69% of participants were interested in receiving free tattoo removal services. Participants were largely non-elderly between the ages of 18–47 years (77%) and 79% were male. Most participants had a history of migration to the U.S., and 67% of participants reported a history of deportation from the U.S. With respect to the risk environment, 60% of participants reported recent drug use, 36% recently injected drugs and 17% recently traded sex. The majority of participants (80%) reported ever being incarcerated in the U.S., Mexico, or both countries. Measures of social exclusion are also reported in Table 1. Approximately one-third of participants (37%) reported having an IFE voter card at the time of interview and 33% were enrolled in Seguro Popular which is Mexico's universal health insurance program. Symptoms of moderate to severe depression were reported by 34% of participants. More than one-half of participants (53%) were recently homeless, 17% were recently denied housing, and 49% reported recently being threatened by local law enforcement. Connections to the labor market were also assessed and 44% reported being recently denied employment, while local social support was low: 67% lacked friends or family in Tijuana.

### Interest in tattoo removal stratified by participant characteristics

We examined interest in tattoo removal by participants' characteristics. There were no statistically significant differences in interest in tattoo removal by age, gender, or U.S. migrant status (Table 1). Similarly, of the risk environment characteristics examined, there were no differences in interest in tattoo removal among those who recently traded sex or were recently incarcerated. Of Social Exclusion variables, interest in tattoo removal did not vary by report of recent homelessness or threats by law enforcement, being denied access to employment, or lacking friends or family in Tijuana. However, participants interested in tattoo removal were less likely than those who were uninterested in tattoo removal to report recent drug use (56 vs. 69%, respectively, *p* = 0.04) and recent injection drug use (32 vs. 45%, respectively, *p* = 0.04). Those interested in tattoo removal services were more likely to have a voter identification card (43 vs. 25%, respectively, *p* < 0.01) and be enrolled in Seguro Popular (37 vs. 25%, respectively, *p* = 0.04) than those uninterested in receiving tattoo removal. Those interested in tattoo removal were more likely to display symptoms of moderate/severe depression (38 vs. 24% among those uninterested in tattoo removal, *p* = 0.02).

### Characteristics of participants' tattoos and stratification by interest in tattoo removal

Participants were asked about the characteristics of their tattoos (Table 2). A minority of participants had only 1 tattoo (27%), 30% reported 2–3 tattoos, and 44% had 4+ tattoos. One-third of participants (37%) had visible tattoos. Tattoo imagery and content varied. Seventy percentage included names or text; animal or nature images (32%), religious (17%), death/skulls (12%), and gang/weapon (8%) tattoos were less commonly reported. Overall, 43% of participants reported disliking some or all of their tattoos; 31% reported experiencing barriers to employment because of their tattoos, and 56% believed that removing their tattoos would help them find employment in Tijuana.

Participants' perceptions of their tattoos and their impact on their lives rather than the characteristics of the tattoos played an important role in participants' interest in removing their tattoos. Specifically, there number or visibility of tattoos or the imagery was not statistically associated with interest in removing the tattoos. Rather, those who reported an interest in tattoo removal were significantly more likely to dislike some or all of their tattoos (60 vs. 6% among those uninterested in tattoo removal, *p* < 0.01), to report barriers to securing employment due to their tattoos (35 vs. 20% of those uninterested in removal; *p* = 0.01), and to believe that tattoo removal would help them find employment (62 vs. 41% among those uninterested in removal, *p* < 0.01).

### Qualitative results: Motivations for seeking tattoo removal

Reasons for desiring tattoo removal varied across participants; themes and illustrative quotes are presented in Table 3. Participants most frequently reported experiencing Employment Barriers (31%): they described a local labor market which wholly rejected tattooed persons. A significant number of participants disliked their tattoos and regretted them (28%). Some participants obtained tattoos as youths or no longer identified with the tattoos or what they represented. Numerous participants felt that tattoos make a bad impression (23%) and contributed to labeling and stereotyping by others. Some participants felt that their tattoos are ugly (19%) and for some participants, this emotion was related to changes in the quality of the image over time. Some participants also believed that their lives would be better without tattoos (16%) and that they negatively impacted participants' mental health (16%), contributing to feelings of discomfort or shame. Other participants identified the toll of discrimination and negative interactions and anxiety/stress resulting from family interactions as motivators for interest in tattoo removal. Negative interactions with law enforcement (7%) were infrequently reported but acknowledged by some participants.

Participants who were uninterested in receiving tattoo removal were asked to explain their reasons and responses fell into three general categories: participants liked their tattoos, or felt that their tattoos are meaningful, or were not interested because they did not have confidence in the results of tattoo removal (Data not shown).

### Factors independently associated with interest in receiving free laser tattoo removal

Table 4 presents results from logistic regression analyses identifying factors independently associated with interest in free tattoo removal services at the study site. No demographic factors were associated with interest in receiving tattoo removal. Of Social Exclusion variables, possession of an IFE Mexican voter identification card was independently associated with being interested in receiving free tattoo removal services at the clinic [Adjusted Odds Ratio (AOR): 2.01; 95% Confidence Interval (CI): 1.04, 3.88; *p* = 0.04], as was reporting moderate to severe depression (AOR: 1.95; 95% CI: 1.03, 3.70; *p* = 0.04). Of Participants' Perceptions regarding the impact of tattoos, believing that tattoo removal would remove barriers to employment (AOR: 2.34; 95% CI: 1.24, 4.42; *p* = 0.01) was also associated with a greater interest in tattoo removal. From Risk Environment variables, drug use in the past 6 months was negatively associated with interest in tattoo removal (AOR: 0.49; 95% CI: 0.26, 0.91; *p* = 0.02).

**Table 4:** Factors independently associated with interest in receiving free laser tattoo removal at the clinic among Mexican adult tattooed free clinic patients (*n* = 278), Tijuana, Mexico, 2013.

	**Adjusted Odds Ratio**	**95% Confidence Interval**	***p*-value**
Migrant status			
Never migrated to U.S.	Reference group	–	
Deported migrant	0.49	0.18, 1.36	0.17
Non-deported US migrant	0.45	0.15, 1.37	0.16
Possesses IFE Mexican voter identification card	**2.01**	**1.04, 3.88**	**0.04**
Seguro popular health insurance	1.41	0.71, 2.78	0.32
Moderate to severe depression	**1.95**	**1.03, 3.70**	**0.04**
Drug use in the past 6 months	**0.49**	**0.26, 0.91**	**0.02**
Has experienced barriers to employment because of tattoos	1.39	0.78, 2.49	0.27
Believes removing tattoos would reduce employment barriers	1.51	0.73, 3.13	0.27
Believes removing tattoos will aid in finding employment in Tijuana	**2.34**	**1.24, 4.42**	**0.01**

Estimates which are significant at p < 0.05 are denoted in bold type.

## Discussion

Tattooing is a pervasive practice worldwide, however, to our knowledge, this study contributes previously unavailable data regarding the perceptions and experiences of a diverse sample of tattooed adults in Mexico. The popular press and several studies have identified tattoo-related stigma and discrimination, including among migrants returning to Mexico (16, 59, 60). Our mixed-methods study corroborates anecdotal evidence with quantitative and qualitative data from a large sample; key findings are contextualized below. This study has important public health implications: laser tattoo removal is desired and can assist tattooed adults engage more broadly with Mexican society and potentially overcome tattoo-related stigma and discrimination (17). Findings may have relevance for other migrant-receiving communities.

For many decades, Mexico has been a major migrant sending country to the U.S. due to diverse social and economic ties that have supported migrant flows between both countries (61, 62). Return migration is not uncommon and our research demonstrates that tattoos are pervasive among persons with histories of U.S. migration. Notably, recent changes to immigration enforcement policies have resulted in the forcible expulsion or voluntary return of Mexican nationals: for example, annually between 2013 and 2020, the United States deported between ~151,000 to ~307,000 migrants to Mexico (12). Consequently, innovative strategies are needed to reintegrate migrants into Mexican society. Moreover, by reducing social exclusion, stigma and discrimination, migrants returning to Mexico may be able to leverage skills and human capital developed in the U.S. for their benefit as well as that of receiving communities in Mexico (63).

In multivariate analyses, participants who believed that removing their tattoos would assist them in finding employment and those possessing a voter identification card were more likely to be interested in tattoo removal. We interpreted these findings to mean that those with a governmental identification card were more likely to be able to navigate the legal aspects associated with interacting with public and private institutions, including the labor market as a governmental identification is required to join workplaces in the formal economy. The qualitative data collected by this study indicated that tattoos often generated stigma and discrimination which contributed to the exclusion of study participants from the labor market. Thus, removal of unwanted tattoos was believed to support access to employment opportunities.

Individuals lacking a Mexican voter identification card are, in effect, undocumented. Studies examining the experiences of undocumented immigrants in the U.S. have demonstrated that lacking a legal status and identification is associated with social stigma, increased stress, anxiety and situations that create vulnerability (64). An undocumented status is also associated with social, economic and health disadvantages due to reduced access to legal, safe and well-paying employment opportunities (65) or public benefits programs (e.g., health insurance coverage) (64). In Mexico, only adults who also possess a birth or naturalization certificate can access the voter card (formerly IFE, now INE card), which serves as the nation's primary form of national identification (66). While Mexico is currently classified as a Upper Middle Income Country (67) it continues to experience economic and geographic disparities; rural communities especially may encounter challenges in providing timely access to birth certificates to their residents, resulting in an “undocumented” status among some individuals (68). Other nations have implemented diverse strategies to overcome challenges to birth certificate access, including birth registration campaigns, providing birth certificates free of charge, and expediting access to this document (68, 69). These strategies may be implemented more broadly to ensure that individuals' identity can be substantiated across the lifespan. Migrants can benefit from Mexico's “*Programa de Repatriación*” (Repatriation Program) and “*Programa Somos Mexicanos*” (We are Mexicans Program) which can help migrants re-establish their identity (*via* access to birth certificates, temporary identification cards and other documents) and obtain critical services early within the repatriation process (e.g., nutrition, health, housing, employment services, and others) (63, 70). To overcome barriers to legal identity among voluntary and forcibly returned migrants, access to the aforementioned programs should be expanded beyond the repatriation period or to other individuals in need of these services.

We observed that participants reporting mental health challenges (i.e., depression symptoms) were more likely to report an interest in tattoo removal while recent substance users were less likely to be interested in tattoo removal. Tattoos may include images that make the individual uncomfortable or embarrassed, hinder their participation in social or economic pro-social activities, or the images may contribute to personal harm due to interpersonal violence (e.g., gang-related motifs) (71). These findings are concordant with extant studies that report that tattoos can provoke adverse mental health impacts when personal identities evolve (e.g., older age) or social affiliations change (e.g., withdrawing from gangs) (14, 39, 72). Entities implementing tattoo removal programs should consider the mental health consequences of having unwanted tattoos when defining the eligibility criteria in order to provide the maximum benefit to persons seeking this service. For example, programs may consider removing all tattoos regardless of their source (e.g., prison; gang affiliation) or location on the body; there is precedent for such an approach in the U.S. and preliminary data indicate that this approach has yielded favorable results for participants (73).

### Limitations

Findings must be considered in light of the following factors. The study may not reflect the experiences of all migrant receiving communities or migrants from other regions. However, the study recruited a large convenience sample of migrants, including tattooed migrants, which are important strengths of this investigation as the research sheds light not only on the experience of tattoos among migrants but how they perceive these marks impact their integration into receiving communities such as Tijuana, Mexico. This study was conducted in 2013 and should be replicated to explore changes in policies, social views regarding tattoos, and changing migration flows from Central America, Eastern Europe and other countries to the US-Mexico border region. Undertaking a similar study in multiple deportee receiving countries would be helpful to understand the challenges faced by deportees seeking to resettle outside of the United States. While our study included a small sample of women, the experiences of tattooed women are generally under-represented in the literature, thus, this study suggests that additional research is needed to understand the challenges that tattooed women may face in Latin America. The data were based on self-report and may be subject to social desirability bias or recall bias. Nevertheless, our study makes significant contributions to the study of social exclusion in the context of Latin America, including the challenges faced by migrants in the region.

## Conclusions

This study explored the issues of tattoo regret, motivations for seeking removal of unwanted tattoos and interest in receiving laser tattoo removal in the U.S.-Mexico border city of Tijuana. Our findings suggest that there is a substantial interest in receiving tattoo removal services, which are quite costly and may be a significant barrier to this service for low income and underserved persons in Mexico (74). A package of services (i.e., governmental identification, tattoo removal) which are publicly financed may assist disadvantaged persons, especially migrants, gain increased access to the labor market, which will aid other life domains (e.g., mental well-being, interactions with law enforcement, income, housing, food insecurity, interpersonal relationships with community members). Similar research should be undertaken elsewhere in order to shed light on the impact of tattoo related stigma across diverse communities and for population subgroups (e.g., forcibly returned migrants, voluntarily returned migrants, asylum seekers, refugees). Findings may shed light on the types of interventions that are needed to overcome tattoo regret and tattoo-related stigma in diverse social context. Longitudinal research is needed to understand whether the social, economic and mental health status of tattooed individuals improves as a result of eliminating unwanted tattoos.

## Data availability statement

The raw data supporting the conclusions of this article will be made available by the authors, without undue reservation.

## Ethics statement

The studies involving human participants were reviewed and approved by University of California, San Diego, Human Research Protection Program; the Ethics Boards of the Health Frontiers in Tijuana Clinic and the Autonomous University of Baja California Medical School. The patients/participants provided their written informed consent to participate in this study.

## Author contributions

VO, JB, and AV-O conceptualized the study and wrote the manuscript. CM and OS assisted with the analyses and preparation of manuscript. All authors prepared and reviewed the manuscript and approved it for submission.

## Funding

This work was supported by the UC GloCal Fellowship and the AIDS International Training Research Program funded by the Fogarty International Center of the National Institutes of Health under Award Numbers R25TW009343 and D43TW008633; the National Institutes of Health National Institute on Drug Abuse under Grant #K01DA025504 and #R37DA019829-S1; the National Institute on Mental Health under Grant #K01MH095680, and the University of California Global Health Institute. The views presented here do not represent the funders.

## Conflict of interest

The authors declare that the research was conducted in the absence of any commercial or financial relationships that could be construed as a potential conflict of interest.

## Publisher's note

All claims expressed in this article are solely those of the authors and do not necessarily represent those of their affiliated organizations, or those of the publisher, the editors and the reviewers. Any product that may be evaluated in this article, or claim that may be made by its manufacturer, is not guaranteed or endorsed by the publisher.
